# Daily Rhythms in Expression of Genes of Hepatic Lipid Metabolism in Atlantic Salmon (*Salmo salar* L.)

**DOI:** 10.1371/journal.pone.0106739

**Published:** 2014-09-03

**Authors:** Mónica B. Betancor, Elsbeth McStay, Matteo Minghetti, Hervé Migaud, Douglas R. Tocher, Andrew Davie

**Affiliations:** Institute of Aquaculture, School of Natural Sciences, University of Stirling, Stirling, Scotland, United Kingdom; Karlsruhe Institute of Technology, Germany

## Abstract

In mammals, several genes involved in liver lipid and cholesterol homeostasis are rhythmically expressed with expression shown to be regulated by clock genes via *Rev-erb 1α*. In order to elucidate clock gene regulation of genes involved in lipid metabolism in Atlantic salmon (*Salmo salar* L.), the orphan nuclear receptor *Rev-erb 1α* was cloned and 24 h expression of clock genes, transcription factors and genes involved in cholesterol and lipid metabolism determined in liver of parr acclimated to a long-day photoperiod, which was previously shown to elicit rhythmic clock gene expression in the brain. Of the 31 genes analysed, significant daily expression was demonstrated in the clock gene *Bmal1*, transcription factor genes *Srebp1*, *Lxr*, *Pparα* and *Pparγ*, and several lipid metabolism genes *Hmgcr*, *Ipi*, *ApoCII* and *El*. The possible regulatory mechanisms and pathways, and the functional significance of these patterns of expression were discussed. Importantly and in contrast to mammals, *Per1*, *Per2*, *Fas*, *Srebp2*, *Cyp71α* and *Rev-erb 1α* did not display significant daily rhythmicity in salmon. The present study is the first report characterising 24 h profiles of gene expression in liver of Atlantic salmon. However, more importantly, the predominant role of lipids in the nutrition and metabolism of fish, and of feed efficiency in determining farming economics, means that daily rhythmicity in the regulation of lipid metabolism will be an area of considerable interest for future research in commercially important species.

## Introduction

Fish are universally acknowledged as a healthy food and are the major source in our diet of the essential and highly beneficial omega-3 (n-3) long-chain polyunsaturated fatty acids (LC-PUFA) [1]. With stagnating or diminishing wild capture fisheries, aquaculture now produces around half the global supply of fish and seafood for human consumption [2]. European aquaculture is dominated by carnivorous species whose natural diets are dominated by protein and lipid with the latter being the prime energy source [3]. Therefore, fish lipid nutrition and metabolism are crucially important issues in aquaculture and, two of the most important issues are the level and source of dietary lipid [4]. Traditionally, dietary lipid was supplied by fish oil, but this is a finite commodity and global supplies are at their sustainable limit and cannot support continued development [5]. As a result, vegetable oils devoid of both omega-3 LC-PUFA and cholesterol are replacing dietary fish oil with potential consequences for both fish health and nutritional quality of the product [6]. Developing a better understanding of the molecular mechanisms controlling lipid metabolism in farmed fish such as Atlantic salmon (*Salmo salar*) will greatly improve the efficiency and sustainability of aquaculture [7].

Daily rhythms in animal behaviour, physiology and metabolism are driven by cell autonomous clocks that are synchronised by environmental cycles, but maintain ≈24 h rhythms even in the absence of environmental cues [8]. While this clock mechanism is central to the canonical “body clock” system there is increasing awareness of the independent actions of localised or “peripheral” clock systems that provide targeted rhythmic control to specific processes. Among the peripheral tissues, rhythmic expression within the liver transcriptome has been an area of particular interest in mammals [9], [10]. In rodents, microarray studies of liver gene expression have indicated that 8–15% of all mRNAs were rhythmically expressed [11], [12], [13], [14]. Many of these mRNAs encoded for enzymes and regulators particularly related to lipid metabolism including fatty acid pathways and cholesterol regulation suggesting such processes were largely regulated by the molecular clock [14].

In mice, a number of genes intrinsically involved in lipid metabolism were shown to be mediated by nuclear receptor subfamily 1, group D, member 1 (REV-ERB 1α; also known as NR1D1) a component of the molecular clock [10]. *Rev-erb 1α* mRNA is rhythmically expressed and subject to regulation by the negative components, Cryptochrome (*Cry*) and Period (*Per*), of the molecular clock [10], [11] as well as by the positive dimer CLOCK/BMAL1 [15]. The REV-ERB 1α protein in turn has the capacity to influence the positive arm of the molecular clock through repression of the BMAL1 transcription factor. REV-ERB 1α is not essential for the cycling of the molecular clock; however it is fundamental in the accuracy and fine-tuning of the clock and has been implicated in adiposity and fatty acid metabolism [10].

The regulation of genes involved in cholesterol and lipid homeostasis by REV-ERB 1α is mediated through sterol regulatory element binding protein (SREBP) pathways and SREBP target genes [10] that include genes of lipid and fatty acid metabolism (*Srebp1*) or cholesterol biosynthesis (*Srebp2*) [16]. *Srebp1* transcription can also be activated through the LXRE response element by liver X receptor (LXR), a nuclear receptor that regulates the metabolism of several important lipids, including cholesterol and bile acids [17]. Other transcription factors that are direct targets of the daily clock in mammals are the peroxisome proliferator-activated receptor-α (PPARα) and PPARγ, which are known to regulate lipid metabolism and energy homeostasis by coordinated actions in a variety of tissues [18].

The cDNAs of transcription factors key to lipid, fatty acid and cholesterol metabolism including *Srebps*, *Ppars* and *Lxr* have been cloned and studied in salmon [19], [20], [21]. Similarly, cDNAs for various clock genes including *Clock, Per1, Per2* and *Bmal1* have been isolated in salmonids [22], [23], [24]. However, whereas the circadian regulation of lipid and cholesterol metabolism and the genes and enzymes involved has been shown in rodents [10], nothing was known of this regulation in teleost fish. The primary aim of the present study was to investigate the relationships between the daily expression of key components of the daily clock and genes of lipid metabolism including the main transcription factors and their target genes in salmon liver. As REV-ERB 1α had been shown in rodents to be a critical factor in daily regulation of SREBP pathways [10], a further aim was to clone the *Rev-erb 1α* cDNA and determine its pattern of expression in a 24 h cycle in salmon.

## Materials and Methods

### Experimental animals and sampling procedures

Stock Atlantic salmon parr (100 fish; mean 24.9±5.4 g, 14.1±0.8 cm) were maintained in a single 1 m^3^ (1000 L) tank at the Niall Bromage Freshwater Research Facilities (Institute of Aquaculture, Stirling, UK). The fish were acclimated to a long day photoperiod cycle (LD 16 h light: 08 h dark) in early March when water temperature was on average 4.6±0.7°C. Feed, EWOS micro parr diet (EWOS Ltd., Bathgate, UK), was offered continuously in excess, during the day and night via a clockwork belt feeder. After 1 month, liver tissue samples were collected from 6 individuals every 4 h over a 24 h period. Briefly, experimental animals were sacrificed via lethal anaesthesia and decapitation. Feeding activity in all animals sampled was confirmed by observing the presence of faecal materials in the intestinal tract. Liver samples were immediately frozen in liquid nitrogen and stored at −70°C until use. A dim red light was used for nocturnal sampling. All experiments were subjected to ethical review by the University of Stirling through the Animal and Welfare and Ethical Review body. The project was conducted under UK Home Office project Licence number PPL 60/03969 in accordance with the amended Animals Scientific Procedures Act implementing EU Directive 2010/63.

### RNA extraction and cDNA synthesis

Approximately 100 mg of liver tissue was homogenised in 1 ml of TRIzol and RNA extracted according to manufacturer’s instructions (Invitrogen, Life Technologies Ltd., Paisley, UK). The resulting RNA pellets were resuspended in MilliQ water to a final RNA concentration of approximately 100 ng/µl. Total RNA concentration was determined by ND-1000 Nanodrop spectrophotometer (Labtech Int., East Sussex, UK). In order to eliminate DNA contamination 5 µg of total RNA was treated with DNase enzyme following DNA-free kit guidelines (Applied Biosystems, Warrington, UK). cDNA was synthesised using 1 µg of DNase-treated RNA and random primers in 20 µl reactions and the High capacity reverse transcription kit without RNase inhibiter according to the manufacturer’s protocol (Applied Biosystems). Final reactions were diluted with DNA/RNA-free water to a final volume of 200 µl. All cDNA samples were stored at −20°C until use in qPCR.

### Identification and cloning of Atlantic salmon *Rev-erb 1α*

*Salmo salar Rev-erb 1α* was cloned as follows: two Atlantic salmon expressed sequence tag clones (Genbank ID: DY724083 and DY731913) were identified by BLAST analysis of published vertebrate *Rev-erb 1α* sequences. 5′ and 3′ ends from the constructed contig were amplified using Rapid Amplification of cDNA Ends (RACE)-PCR with the RACE cDNAs generated from 1 µg of salmon whole brain total RNA using the SMART RACE kit as described in the user manual (Clontech, Mountain View, CA). The 5′ and 3′ RACE amplicons were generated by two rounds of PCR using *Rev-erb 1α 1* 5′R1 and *Rev-erb 1α* 5′R2 primers or *Rev-erb 1α* 3′F1 and *Rev-erb 1α* 3′F2, respectively (Table S1). The final full-length sequence was confirmed by two rounds of PCR using nested primers designed to amplify end-to-end full-length cDNAs (REV-ERB 1α_full_F1: REV-ERB 1α_full_R1 and REV-ERB 1α_full_F2: REV-ERB 1α_full_R2). All PCRs were run at annealing temperatures as listed in Table S1 with an extension time of 1 min/Kb of predicted PCR product, with 3 min applied for unpredictable RACE PCR products. All primers were designed using Primer Select Ver. 6.1 program (DNASTAR, www.dnastar.com). Sequencing was performed using a Beckman 8800 autosequencer and Lasergene SEQman software (DNASTAR) used to edit and assemble DNA sequences.

### Quantitative real-time PCR (qPCR)

With the exception of *Rev-erb 1α*, all qPCR assays were established and verified previously [21], [22], [25]. In order to determine diel patterns of gene expression, qPCR was carried out on clock genes *Bmal 1*, *Clock*, *Per1*, *Per2* and *Rev-erb 1α*; lipid-associated transcription factor genes, *Srebp1, Srebp2, Lxr, Pparα* and *Pparγ*; and key genes involved in major lipid pathways including fatty acid synthesis (*Fas, D6Fad, D5Fad, Elovl5* and *Elovl2*) and catabolism (*Cpt1* and *Aco*), cholesterol metabolism (*Hmgcr*, *Mev*, *Dhcr7*, *Ipi*, *Abca1* and *Cyp71α*) and lipoprotein metabolism (*Apoa1*, *Apob*, *ApocII*, *Ldlr*, *El*, *Lpla*, *Lplb* and *Lplc*) (Table S2). qPCR primer sequences and annealing temperatures are described in Table S3. All samples were run in duplicate and assays were performed as follows, 95°C for 15 min and 45 cycles of 95°C for 15 s, anneal for 15 s and 72°C for 30 s. This was followed by a temperature ramp from 70–90°C for melt-curve analysis to verify that no primer–dimer artefacts were present and only one product was generated from each qPCR assay. Quantification was achieved by a parallel set of reactions containing standards consisting of serial dilution of spectrophotometrically-determined, linearised plasmid containing partial cDNA sequences.

### qPCR normalisation and statistical analysis

GeNorm analysis was carried out on three potential housekeeping genes over the long day liver diel profile to determine the most stable and appropriate reference gene for this tissue. Of the three genes studied (*β-actin, EF-α* and *GAPDH*) analysis highlighted elongation factor α (*EF-α)* as the most appropriate housekeeping gene with the greatest stability (M value = 0.9).

Analysis of Variance (ANOVA) was used to determine significant effects of time and Tukey’s test was used to determine the significance of differences between sample time points and mean of different sample sets (InStat 3.1, Graphpad software inc.). Data were then fitted to a cosine wave in order to determine the presence of a significant daily rhythm. Raw data was analysed using Acro circadian analysis programs (University of South Carolina, USA; http://www.circadian.org/softwar.html). Acro analysis determines both the significance, acrophase (peak in expression) mean and amplitude of raw data using the equation:
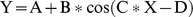
where Y is level of gene expression as a percentage of the mean, A is the baseline, C is the frequency multiplier (set to fixed period of 24 h), and D is the acrophase (peak time of the cosine approximation) of the data set [22]. A significant daily rhythm was deemed present when *p* value was less than 0.05 for all statistical analysis. All results are presented as % of mean expression whereby each normalized copy number value was converted to a % of the average total copy number of the gene of interest. Data were then presented as mean ± SEM in relation to zeitgeber time (ZT) whereby ZT 00∶00 occurred at lights on and lights off was at ZT 16∶00.

## Results

### Salmon REV-ERB 1α sequence and phylogenetics

A 2987 bp full-length cDNA sequence (Accession number: 1714461) was obtained by several runs of 5′ and 3′ RACE PCR. This contained an open reading frame (ORF) of 1824 bp encoding a putative protein of 608 amino acids and 3′ and 5′ untranslated regions (UTR) of 349 bp and an 814 bp, respectively. Atlantic salmon putative REV-ERB 1α possessed a ligand binding domain, and the predicted DNA binding domain composed of two C4-type zinc fingers, each one containing a group of four coordinating cysteine residues, as well as one crystal form similar to human 1HLZ-A [26] (Fig. S1). Alignment and phylogenetic analysis of the deduced amino acid sequence of salmon *Rev-erb 1α* in relation to other previously characterised vertebrate *Rev-erb 1α* and *Rev-erb 1β* sequences showed clustering in two different clades containing mammalian REV-ERB 1α and β (Fig. S2). Homologous proteins exist in other fish species such as tilapia (*Oreochromis niloticus*) and fugu (*Takifugu rubripes*), with the Atlantic salmon protein displaying 57% and 71% identities with human and zebrafish (*Danio rerio*) *Rev-erb 1α* sequences, respectively.

### Clock gene expression in liver

**Figure 1:**
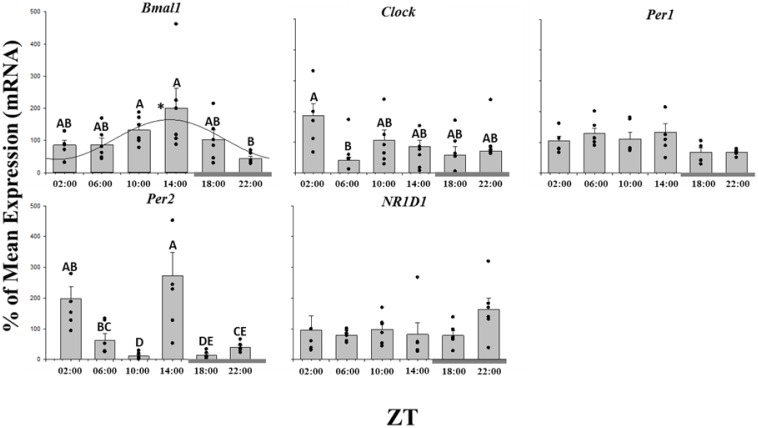
Twenty-four hour expression profiles of clock genes in the liver of salmon parr acclimated to LD photoperiod. Results are displayed in relation to Zeitgeiber time (ZT), where ZT 0 is the onset of light. Gene expression data is displayed as the percentage of the mean ± SEM and includes the spread of the data. The presence of a cosine wave denotes a significant circadian rhythm by acro analysis. The grey bar at the bottom of the graph represents the dark period. The presence of different letters represents statistically significant differences between samples by ANOVA and Tukey’s test (P<0.05).

Of the five clock genes investigated all were expressed in the liver, but only *Bmal 1* displayed a significant daily pattern of expression when results were fitted to a cosine wave using Acro analysis (Fig. 1; Table 1) [27]. The acrophase of *Bmal1* occurred at 3 h prior to lights off at ZT 13∶00±3.9.

**Table 1:** P value of 24 h profiles of gene expression Acro and ANOVA analysis and acrophase where significant rhythm is present.

Gene	Acro (p value)	Acrophase ZT	ANOVA (p value)
***Bmal1***	<0.05	13∶00±3.09	<0.05
***Clock***	n.s.	-	<0.05
***Per 1***	n.s.	-	n.s.
***Per 2***	n.s.	-	<0.05
***Rev-erb 1α***	n.s.	-	n.s.
***Lxr***	<0.05	13∶00±2.73	n.s.
***Srebp1***	<0.05	13∶00±2.41	<0.05
***Srebp 2***	n.s.	-	n.s.
***Pparα***	<0.05	13∶00±2.26	<0.05
***Pparγ***	<0.05	9∶00±2.33	<0.05
***Hmgcr***	<0.05	9∶00±3.12	<0.05
***Mev***	n.s.	-	n.s.
***Ipi***	<0.05	9∶00±2.57	n.s.
***Dhcr7***	n.s.	-	<0.05
***Abca1***	n.s.	-	n.s.
***Cyp71α***	n.s.	-	n.s.
***D5Fad***	n.s.	-	n.s.
***D6Fad***	n.s.	-	n.s.
***Elovl2***	n.s.	-	n.s.
***Elovl5a***	n.s.	-	n.s.
***Fas***	n.s.	-	n.s.
***Aco***	n.s.	-	n.s.
***Cpt1***	n.s.	-	n.s.
***ApoA1***	n.s.	-	n.s.
***ApoB***	n.s.	-	n.s.
***ApoCII***	<0.05	9∶00±2.97	n.s.
***Ldlr***	n.s.	-	n.s.
***El***	<0.05	21∶00±3.23	n.s.
***Lpla***	n.s.	-	n.s.
***Lplb***	n.s.	-	n.s.
***Lplc***	n.s.	-	n.s.

n.s. denotes no statistical differences between the different sampling points. Acrophases (circadian peak times) were calculated by non-linear regression fit of a cosine function. Data are expressed as acrophase ±95% confidence intervals.

### Transcription factor gene expression

**Figure 2:**
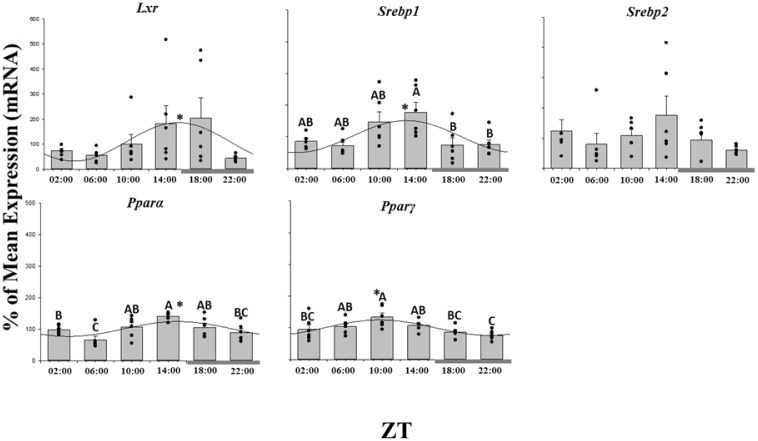
Twenty-four hour expression profiles of transcription factors involved in the regulation of lipid metabolism in the liver of salmon parr acclimated to LD photoperiod. Results are displayed in relation to Zeitgeiber time (ZT), where ZT 0 is the onset of light. Gene expression data is displayed as the percentage of the mean ± SEM and includes the spread of the data. The presence of a cosine wave denotes a significant circadian rhythm by acro analysis. The grey bar at the bottom of the graph represents the dark period. The presence of different letters represents statistically significant differences between samples by ANOVA and Tukey’s test (P<0.05).

Of the studied metabolism-related genes, those belonging to the transcription factors presented the most consistent rhythmicity. Thus, four of the five studied transcription factor genes presented 24 h rhythmic expression patterns in the liver of LD Atlantic salmon (Fig. 2; Table 1). *Lxr*, *Srebp1*, *Pparα* and *Pparγ* exhibited significant rhythmic variations in mRNA expression, whereas *Srebp2* did not show such a tendency (Fig. 2). However, for *Lxr* unlike *Srebp1*, *Pparα* and *Pparγ* while expression was rhythmic there were no significant temporal differences in expression levels. The acrophase of expression cycles was comparable for *Lxr*, *Srebp1* and *Pparα* but 4 h advanced for *Pparγ* (Table 1).

### Lipid and cholesterol homeostasis gene expression

**Figure 3:**
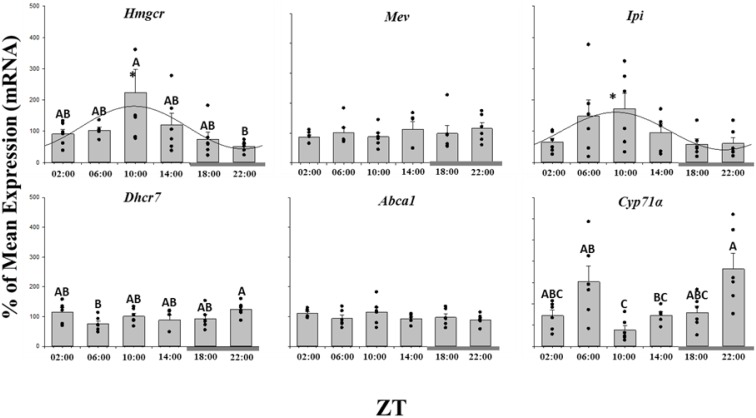
Twenty-four hour expression profiles of genes related to cholesterol metabolism in the liver of salmon parr acclimated to LD photoperiod. Results are displayed in relation to Zeitgeiber time (ZT), where ZT 0 is the onset of light. Gene expression data is displayed as the percentage of the mean ± SEM and includes the spread of the data. The presence of a cosine wave denotes a significant circadian rhythm by acro analysis. The grey bar at the bottom of the graph represents the dark period. The presence of different letters represents statistically significant differences between samples by ANOVA and Tukey’s test (P<0.05).

**Figure 4:**
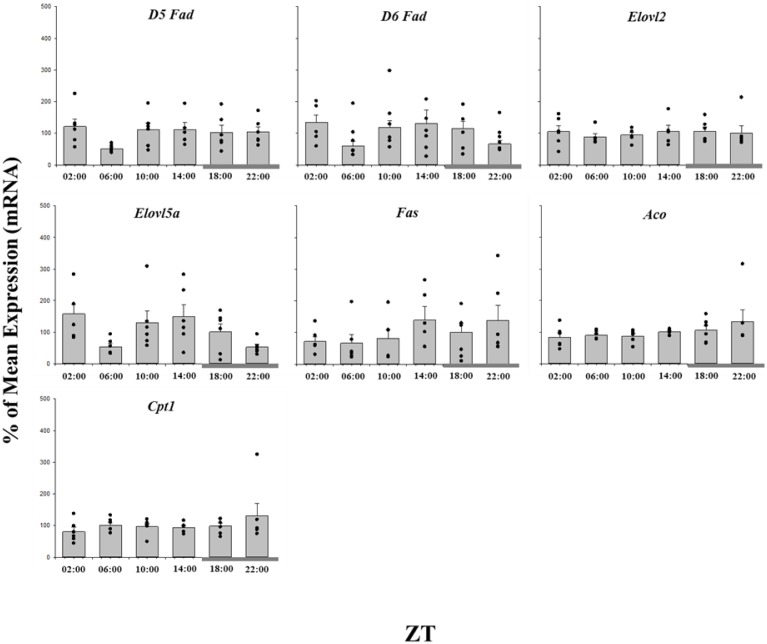
Twenty-four hour expression of fatty acid metabolism genes in the liver of salmon parr acclimated to LD photoperiod. Results are displayed in relation to Zeitgeiber time (ZT), where ZT 0 is the onset of light. Gene expression data is displayed as the percentage of the mean ± SEM and includes the spread of the data. The presence of a cosine wave denotes a significant circadian rhythm by acro analysis. The grey bar at the bottom of the graph represents the dark period. The presence of different letters represents statistically significant differences between samples by ANOVA and Tukey’s test (P<0.05).

**Figure 5:**
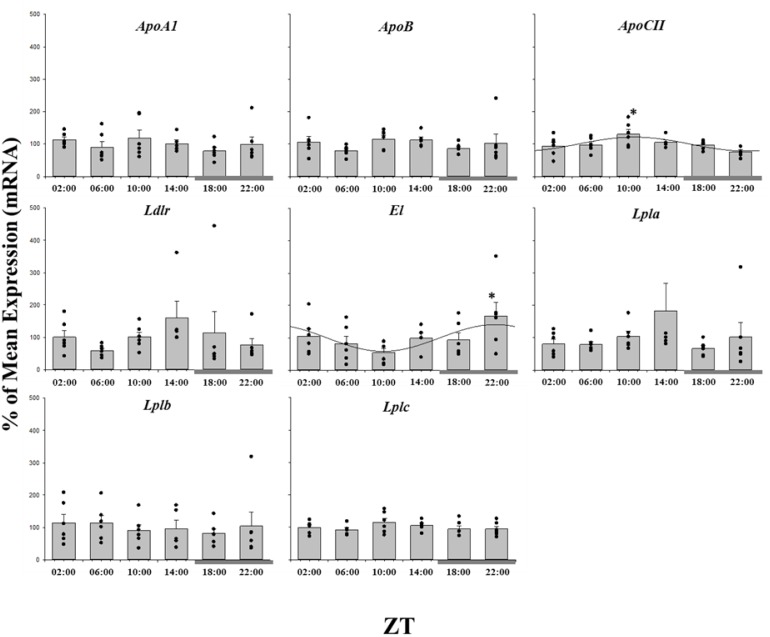
Twenty-four hour expression of lipoprotein metabolism genes in the liver of salmon parr acclimated to LD photoperiod. Results are displayed in relation to Zeitgeiber time (ZT), where ZT 0 is the onset of light. Gene expression data is displayed as the percentage of the mean ± SEM and includes the spread of the data. The presence of a cosine wave denotes a significant circadian rhythm by acro analysis. The grey bar at the bottom of the graph represents the dark period. The presence of different letters represents statistically significant differences between samples by ANOVA and Tukey’s test (P<0.05).

The daily expression profiles of the cholesterol metabolism genes showed that only *Ipi* and *Hmgcr* displayed a significant daily rhythm, showing peak expression around ZT 09∶00, seven hours before the dark phase (Fig. 3; Table 1). Whilst *Dhcr7* did not display a significant daily rhythm, significant differences in expression between time points was observed, with maximal transcript abundance at ZT 22∶00, at the end of the dark phase (Fig. 3). None of the genes involved in fatty acid synthesis or catabolism displayed a significant daily profile of expression, or any significant differences among the time points (Fig. 4; Table 1). Of the lipoprotein metabolism genes, *ApoCII* and *El* followed a rhythmic expression, reaching their acrophase at ZT 10∶00 and 22∶00 respectively, although there were no significant temporal differences in expression levels (Fig. 5).

## Discussion

While in mammals the interaction of the clock gene mechanism and liver transcriptome had been established, the importance of daily regulation on diverse metabolic processes in fish remained to be determined. The results of the present study demonstrated, for the first time, daily regulation of specific genes of lipid metabolism and homeostasis in liver of Atlantic salmon, and provided an insight into the molecular control mechanisms involved.

The molecular clock mechanism is based on an autoregulatory feedback loop that takes approximately 24 h to complete and synchronises a multitude of molecular, physiological and behavioural process to the daily 24 h cycle [8]. As in other tissue types, the hepatic oscillator is believed to be centred on two transcriptional-translational activators, BMAL1 and Clock, and two classes of repressors, Period and Cryptochrome [15]. In the present study the expression of four core clock genes, *Bmal1, Clock, Per 1* and *Per 2* was characterised, with *Bmal1* the only one to display a significant daily profile of expression. Such expression was previously observed in zebrafish and gilthead sea bream (*Sparus aurata*) liver [28], [29] and the salmonid brain [22], [30]. In addition to its role in the control of daily rhythm, BMAL 1 has been suggested to contribute to lipid metabolism control and adipogenesis in mammals [31]. It has also been shown to regulate rhythmic gene expression throughout the liver transcriptome [32], [33]. However the daily profile of *Bmal1* expression identified in this study, particularly in the absence of additional rhythmic expression of other clock genes, may indicate an additional function of *Bmal1* in the liver of the Atlantic salmon. Interestingly *Bmal1* itself can be regulated by elements of lipid metabolism pathways. Specifically PPARα has been shown to control *Bmal1* expression in the liver *via* direct binding to a PPRE located in the *Bmal1* promoter [34], [35]. With regard to the present investigation, mRNA expression of *Bmal1* and *Pparα* in the liver appeared to be in phase with their acrophases occurring at 13∶00±3.09 and 13∶00±2.73, respectively, indicating that expression and regulation of these genes may be related as in mammals. In addition, *Bmal1* is a direct target of vascular *Pparγ* [36] although its role in the regulation of the clock gene *Bmal1* is unknown in the liver.

In contrast to previous studies on teleost liver [28], [29], [37], [38], *Clock*, *Per1* and *Per2* did not display oscillatory patterns in a 24 h cycle, however, the results for *Clock* and *Per2* are consistent with those previously obtained in the brain of Atlantic salmon [26]. While in the teleost brain light dark cycle is considered to be the primary zeitgeber [39], hepatic clocks appear to demonstrate a greater variety in entrainment pathways [32]. Both light as well as food availability can act as a potential entraining signal [29], [40]. Equally in mammals it has been demonstrated that the phase of the daily clock of the liver can be altered by feeding time, decoupling it from the unaltered rhythm of the suprachiasmatic nucleus (SCN), which is the central daily pace maker in mammals [41]. Due to the liver being fundamental to the metabolism and overall health and welfare of an organism it is hypothesised that in both mammals and teleosts the hepatic clock can be decoupled from central clock mechanisms and non-photic entrainment allows adaptation to immediate environmental changes such as food availability [29], [32].

With regard to the temporal regulation and expression of the liver lipid metabolism it was necessary to determine if *Rev-erb 1α,* a key connection between the molecular clock and liver lipid metabolism in mammals, played a similar role in Atlantic salmon. Alignment and phylogenetic analysis showed that the sequence identified displayed considerable similarity to other vertebrate *Rev-erb 1α* sequences, and presented homologous predicted structural components with human REV-ERB 1α protein [26]. In contrast to mammals, analyses of *Rev-erb 1α* expression over the 24 h period revealed no temporal differences in levels of mRNA expression in the salmon liver. This finding does not discount regulation of protein as opposed to transcript level or the potential presence of additional copies or homologs of *Rev-erb 1α*. Salmonids have undergone two further genome duplication events in comparison to mammals [42] yet the relevance of this to the clock system is unknown.

One of the most interesting findings of the present study was the strong rhythmicity displayed by the transcription factors. Of the 31 genes examined in the present study, 9 followed a rhythmic pattern in Atlantic salmon liver, with transcription factors representing the most consistent group (four out of five genes). Although precisely how the circadian clock acts to control metabolic rhythms is still unclear, for instance, *Rev-erb 1α* was shown to regulate the expression of *Srebps* and its target genes [10]. However, rhythmic expression of *Srebp1* was observed in the salmon liver despite the lack of rhythmic expression of *Rev-erb 1α,* which may infer protein level or non- *Rev-erb 1α* regulation of the pathway. Although *Srebp1* target genes did not similarly display significant rhythmic expression, the peak expression of *Srebp1* at ZT 14∶00 was coincidental with higher expression of several genes including *D6 Fad*, *Elovl5a*, *Fas*, *Cpt1* and *Aco,* all known to be target genes of *Srebp1* in mammals [43].

The PPARs family members are known to regulate lipid metabolism and energy homeostasis in several teleost species [20], [44], [45]. However, in fish it was not as clear whether *Ppar* expression showed rhythmic variation as described for mammals [9] and, similarly, there was limited information regarding temporal fluctuations of lipid metabolism genes over the daily LD cycle. The present study showed that both *Pparα* and *γ* expression followed a rhythmic pattern, with *Pparα* reaching a peak at ZT 14∶00 concurrently with *Srebps*, whereas *Pparγ* peaked at ZT 9∶00 which contrasts to the results of Huang et al. [37], but is in agreement with Paredes et al. [46]. In the present study the difference in acrophase could be correlated with the known functions of PPARs, with α expression being high under low feeding conditions, just prior to the scotophase, whereas γ expression was highest when lipid levels would be high in the middle of the light phase. It is important to note that salmonids are visual feeders, thus it is expected a decrease in the feed intake at the scotophase [47]. Similarly, the rhythmic expression observed in *El* and *ApoCII*, two genes involved in lipoprotein metabolism, appeared to be linked to lipid availability. Thus, an increase in the expression of *El*, a phospholipid and triacylglycerol hydrolysing enzyme, was observed during the dark phase, a period when feeding is low and release of lipid from lipoproteins is likely required. In contrast, *ApoCII*, which is important in the formation of very-low density lipoproteins, is higher at the middle of the light phase when there is greatest feeding and thus high plasma lipid levels promoting liver lipoprotein production. The physical drivers of rhythmic expression in both the clock genes as well as lipid metabolism remain to be determined, however it is likely that either photoperiod, feed availability or an interaction of the two will control the expression patterns observed. Interestingly in sea bream, while Vera et al. [29] reported feeding patterns to be the driver of liver clock gene rhythms, Paredes et al. [46] concluded that photoperiod was the strongest regulator of liver lipid metabolism cycles.

Another transcription factor that displayed a significant daily rhythm was *Lxr*, which reached its zenith at dusk when both *Srebp1* and *2* were at their nadir. Mammalian *Lxrα* is activated by binding oxysterols (cholesterol metabolites) arising from increased intracellular cholesterol [48], [49]. This suggests that the increase in *Lxr* transcript could be related to the daily rhythm of cholesterol biosynthesis in the salmon liver, displaying higher *de novo* production during the dark phase when there is a fasting state, as reported for humans [50].

In addition, when intracellular levels of cholesterol are low, SREBPs are cleaved and released to act as transcription factors. In mammalian systems SREBPs accumulate in the nucleus and trigger the synthesis of HMG-CoA reductase (*Hmgcr*), the rate-limiting enzyme in cholesterol biosynthesis [10]. In salmon liver, *Hmgcr* acrophase was reached at ZT 10∶00, whereas *Srebp1* acrophase occurred three hours later, inconsistent with *Hmgcr* being a *Srebp* target gene in this species. In agreement with the present data, recent studies, both *in vivo* and *in vitro*, have shown that this pathway is likely to be regulated differently in salmon compared to mammals [7], [21]. In contrast, however, negative regulation appeared to exist between *Hmgcr* and *Lxr*, as similarly described for mammals [36], when one gene reaches its zenith the other is at its lowest expression level.

In mammals, the mechanisms involved in the conversion of cholesterol to bile acid appear to be under a degree of daily regulation via REV-ERB 1α [10]. However, in Atlantic salmon liver this was not the case, suggesting that regulation may be at the protein level as opposed to transcriptional level or a differential pathway as previously described in mammals may be in place. Isopentenyl diphosphate isomerase (*Ipi*), an enzyme in the cholesterol synthesis pathway from mevalonate pyrophosphate to farnesyl pyrophosphate, displayed a rhythmic expression, reaching its peak at around ZT 9∶00. This enzyme did not follow the tendency observed by *Srebps*, *Lxr* or *Cyp71a*, but it must be noted that each one of the synthetic steps is under rigid negative feedback regulation by some intermediate substrates and not only by the final product, cholesterol. This could also explain why the other cholesterol metabolism genes *Mev*, *Dhcr7* and *Abca1* show different expression profiles, depending on which step is being promoted at that moment.

Overall the current study provided clear evidence for the conservation of rhythmic daily regulation of liver lipid metabolism in teleosts as has been reported in mammals [14]. However it is the practical significance of this study that warrants further research as understanding the mechanisms involved in the regulation of absorption, transport and metabolism of lipids and fatty acids is of increasing importance in aquaculture [21]. The current work is the first evidence that Atlantic salmon lipid metabolism is under the influence of environmental parameters that are routinely manipulated in culture for other production reasons e.g. photoperiod management of sexual maturation [51]. As such, it is suggested that the possibility of environmental manipulation to optimise lipid metabolism in farmed salmon should be further explored.

In conclusion, the current study has provided the first evidence for the daily expression of genes involved in cholesterol and lipid homeostasis in the liver of Atlantic salmon under an LD cycle. Transcription factors appear to present a strong rhythmic expression, which could indicate their role as synchronisers. However, the expression of some target genes did not display a significant daily expression, denoting that this activation pathway may differ among mammals and teleosts or that other entraining factors may regulate their expression in liver. These findings provide the basis towards understanding the role of the circadian clock in the regulation of lipid metabolism in teleost fish and provide a novel approach to improve fish aquaculture by optimising feeding protocols and/or environmental conditions to match fish lipid metabolism rhythms.

## Supporting Information

Figure S1 **Alignment of the deduced protein sequence for Atlantic salmon REV-ERB 1α along with tilapia, zebrafish, as well as human.** The conserved amino acids are shaded in grey. Predicted DNA binding domain (top) and ligand binding domain (bottom) identified using a CDD search [52] are boxed. The structurally coordinating cysteine residues belonging to the two C4 Zinc fingers are identified with an asterisk.
(TIF)

Figure S2 **Phylogenetic analysis of the deduced amino acid sequence for REV-ERB 1α in relation to other vertebrate REV-ERB 1α and REV-ERB 1β sequences.** The evolutionary history was inferred using the neighbour-joining method [53]. The percentage of replicate trees in which the associated taxa clustered together in the bootstrap test (1000 replicates) are shown next to the branches [54]. The evolutionary distances were computed using the maximum composite likelihood method [55] and are presented as the number of base substitutions per site. Phylogenetic analyses were conducted in MEGA5 [55]. GenBank Accession Numbers: zebrafish REV-ERB 1α (NP_991292.1), tilapia REV-ERB 1α (XP_003442479.1), zebra mbuna REV-ERB 1α (XP_004556567.1), sheep REV-ERB 1α (NP_001124501.1), human REV-ERB 1α (NP_068370.1), mouse REV-ERB 1α (NP_663409.2), tilapia REV-ERB 1β (XP_005459783.1), fugu REV-ERB 1β (XP_003969152.1), zebrafish REV-ERB 1β (NP_001092087.1), mouse REV-ERB 1β (NP_035714.3) and human REV-ERB 1β (NP_001138897.1).
(TIF)

Table S1 **Abbreviation, full name and function of all genes investigated.**
(DOCX)

Table S2 **Primer pairs and sequences for** ***Rev-erb1α*** **identification including primer name, purpose, sequence and annealing temperature.**
(DOCX)

Table S3 **Primers used for qRT-PCR.**
(DOCX)
