# Supplementary figures and images for: Daily Rhythms in Expression of Genes of Hepatic Lipid Metabolism in Atlantic Salmon (Salmo salar L.)
Source: PLoS One. 2014 Sep 3;9(9):e106739. doi: 10.1371/journal.pone.0106739 (PMC4153669; doi:10.1371/journal.pone.0106739)

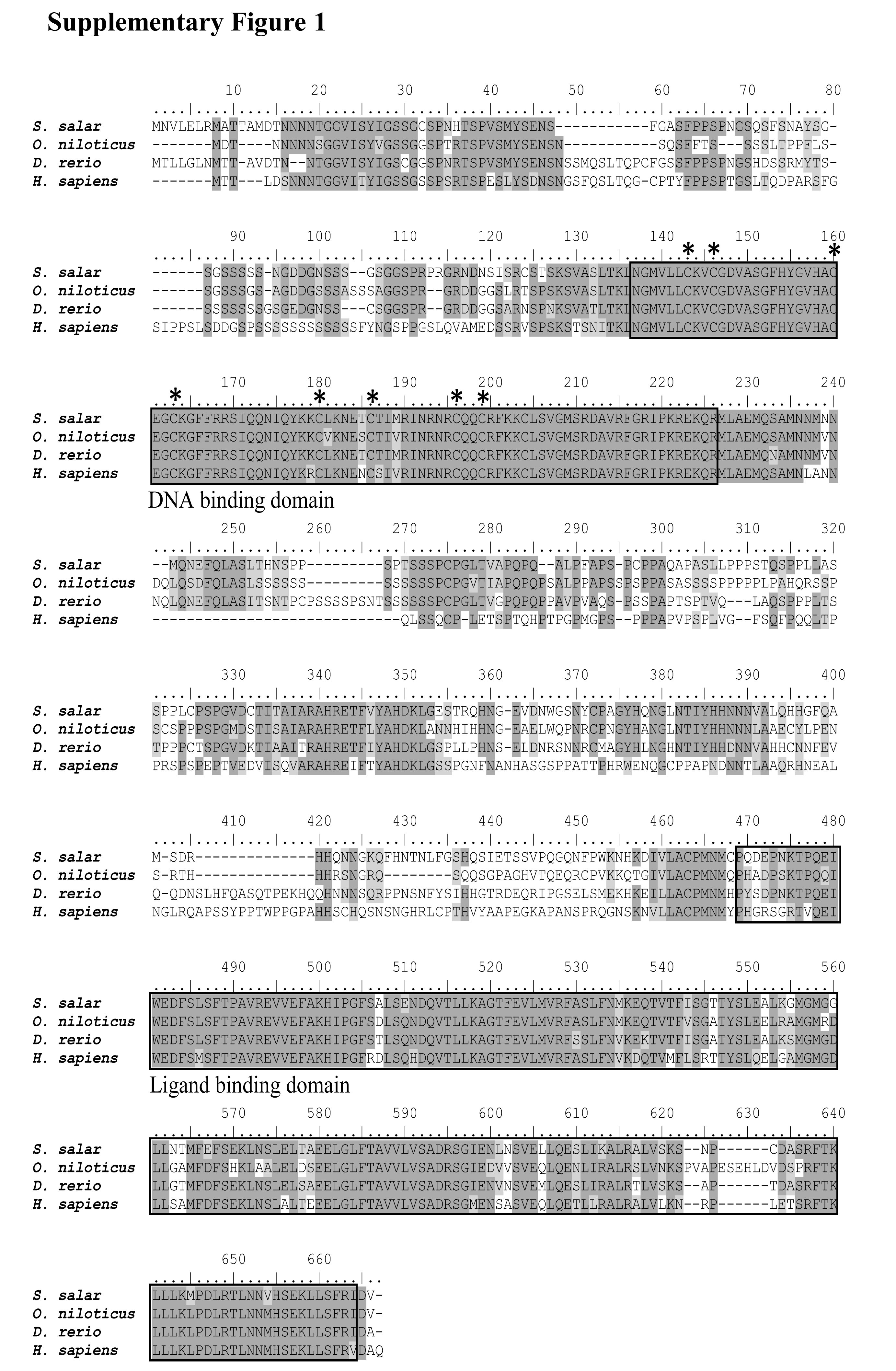

Supplement: Figure S1 — Alignment of the deduced protein sequence for Atlantic salmon REV-ERB 1α along with tilapia, zebrafish, as well as human. The conserved amino acids are shaded in grey. Predicted DNA binding domain (top) and ligand binding domain (bottom) identified using a CDD search [52] are boxed. The structurally coordinating cysteine residues belonging to the two C4 Zinc fingers are identified with an asterisk. (TIF) [file pone.0106739.s001.tif]

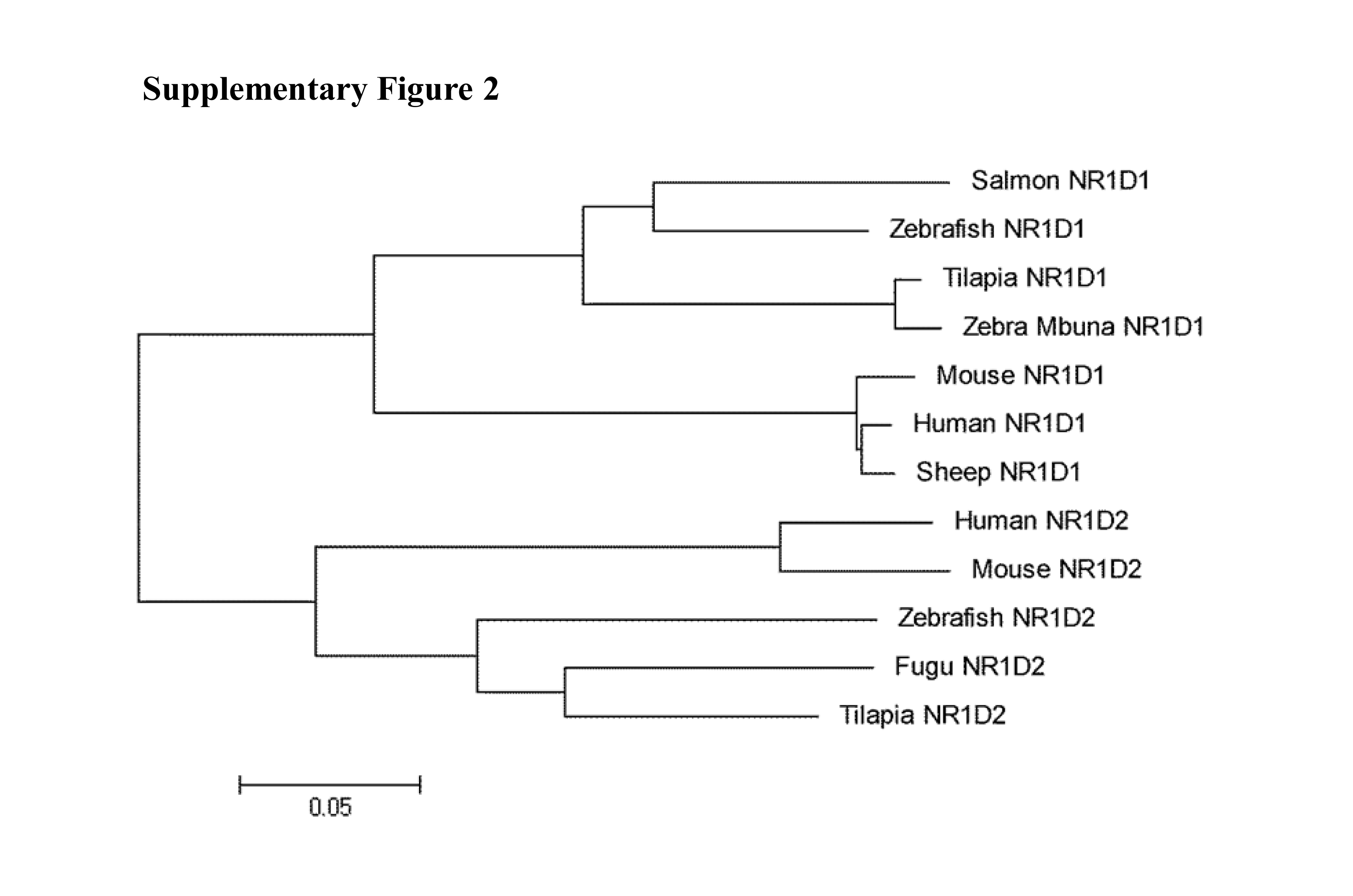

Supplement: Figure S2 — Phylogenetic analysis of the deduced amino acid sequence for REV-ERB 1α in relation to other vertebrate REV-ERB 1α and REV-ERB 1β sequences. The evolutionary history was inferred using the neighbour-joining method [53]. The percentage of replicate trees in which the associated taxa clustered together in the bootstrap test (1000 replicates) are shown next to the branches [54]. The evolutionary distances were computed using the maximum composite likelihood method [55] and are presented as the number of base substitutions per site. Phylogenetic analyses were conducted in MEGA5 [55]. GenBank Accession Numbers: zebrafish REV-ERB 1α (NP_991292.1), tilapia REV-ERB 1α (XP_003442479.1), zebra mbuna REV-ERB 1α (XP_004556567.1), sheep REV-ERB 1α (NP_001124501.1), human REV-ERB 1α (NP_068370.1), mouse REV-ERB 1α (NP_663409.2), tilapia REV-ERB 1β (XP_005459783.1), fugu REV-ERB 1β (XP_003969152.1), zebrafish REV-ERB 1β (NP_001092087.1), mouse REV-ERB 1β (NP_035714.3) and human REV-ERB 1β (NP_001138897.1). (TIF) [file pone.0106739.s002.tif]
