# Supplementary material for: Daily Rhythms in Expression of Genes of Hepatic Lipid Metabolism in Atlantic Salmon (Salmo salar L.)
Source: PLoS One. 2014 Sep 3;9(9):e106739. doi: 10.1371/journal.pone.0106739 (PMC4153669; doi:10.1371/journal.pone.0106739)
Supplement: Table S1 — Abbreviation, full name and function of all genes investigated. (DOCX) [file pone.0106739.s003.docx]

**Supplementary Table 1**.- Abbreviation, full name and function of all genes investigated

| **Gene** | **Full Name** | **Gene Function** |
| --- | --- | --- |
| ***Bmal1*** | *Brain and muscle aryl hydrocarbon receptor nuclear translocator (ARNT)-like* | Forms part of the positive arm the circadian molecular clock. BMAL forms hetrodimer with CLOCK and initiates the transcription of *Per* and *Cry* genes. |
| ***Clock*** | *Circadian Locomotor Output Cycles Kaput* | In conjunction with BMAL forms the positive arm of the circadian clock and regulates negative elements *Cry* and *Per*. |
| ***Per 1*** | *Period1* | In conjunction with Cry genes forms negative components of the circadian clock. |
| ***Per 2*** | *Period2* | In conjunction with Cry genes forms negative components of the circadian clock. |
| ***Rev-erb 1α*** | Nuclear receptor subfamily 1, group D, member 1 | NR1D1 is regulated in a circadian manner by BMAL and via E-box elements regulates a number of clock controlled genes. |
| ***Srebp1*** | *Sterol Regulatory Element-Binding Protein 1* | Indirectly involved in cholesterol synthesis. In mammals involved in sensing cholesterol availability in the ER |
| ***Srebp 2*** | *Sterol Regulatory Element-Binding Protein 2* | Indirectly involved in cholesterol synthesis by regulating *Hmgcr.* |
| ***Lxr*** | *Liver x receptor* | Regulates cholesterol and fatty acids by activating Cyp7a , the rate limiting enzyme in the conversion of cholesterol to bile acid. |
| ***Pparα*** | *Peroxisome proliferator-activated receptor alpha* | Role in promoting hepatic fatty acid oxidation and ketogenesis in response to fasting. |
| ***Pparγ*** | *Peroxisome proliferator-activated receptor gamma* | Activates transcriptional programs for lipid storage and lipogenesis. |
| ***D6Fad*** | *D6 – Fatty acid desaturase* | Required for the synthesis of highly unsaturated fatty acids. |
| ***D5Fad*** | *D5 – Fatty acid desaturase* | Required for the synthesis of highly unsaturated fatty acids. |
| ***Elovl2*** | *Elongation of very long chain fatty acids protein 2* | Participates in the biosynthesis of long chain poly unsaturated fatty acids. Elongation of C22. |
| ***Elovl5a*** | *Elongation of very long chain fatty acids protein 5* | Participates in the biosynthesis of long chain poly unsaturated fatty acids. Primarily the elongation of C18 and C20. |
| ***Fas*** | *Fatty acid synthase* | Corresponding protein catalyzes fatty acid synthesis |
| ***Hmgcr*** | *3-hydroxy-3-methyl-glutaryl-CoA reductase* | Resulting HMGCoA reductase enzyme is the rate limiting enzyme in cholesterol synthesis. |
| ***Mev*** | *Mevalonate kinase* | Catabolizes mevalonate to mevalonate-5P |
| ***Dhcr7*** | *D-7 dehydrocholesterol reductase* | Catalyzes the conversion of 7-dehydrocholesterol to cholesterol. |
| ***Ipi*** | *Isopentenyl diphosphate isomerase* | Isomerase that catalyzes the conversion of the relatively un-reactive isopentenyl pyrophosphate to the more reactive electrophile dimethylallyl pyrophosphate |
| ***Abca1*** | *ATP-binding cassette, subfamily A, member 1* | Key gatekeeper influencing intracellular cholesterol transport |
| ***Cyp71α*** | *Cholesterol 7a-hydroxylase 1 alpha* | Rate limiting enzyme in bile acid synthesis |
| ***Cpt1*** | *Carnitine palmitoyltransferase I* | Mitochondrial enzyme that mediates the transport of long-chain fatty acids across the membrane by binding them with carnitine |
| ***Aco*** | *Acyl-CoA oxidase* | Oxidoreductase that participates in β-oxidation |
| ***ApoA1*** | *Apolipoprotein A1* | Major apolipoprotein of high-density lipoproteins that mediates cholesterol removal through the action of Abca1 |
| ***ApoB*** | *Apolipoprotein B* | Primary apolipoprotein of chylomicrons and LDL |
| ***ApoCII*** | *Apolipoprotein CII* | Component of very low density lipoproteins and chylomicrons |
| ***Ldlr*** | *Low-density lipoprotein receptor* | Its activation leads to an increase in cellular cholesterol uptake |
| ***El*** | *Endothelial lipase* | Enzyme that catabolizes HDL-cholesterol |
| ***Lpla*** | *Lipoprotein lipase a* | Hydrolizes triglicerides in lipoproteins such as those found in chylomicrons and very low-density lipoproteins |
| ***Lplb*** | *Lipoprotein lipase b* |  |
| ***Lplc*** | *Lipoprotein lipase c* |  |
