# Supplementary material for: Daily Rhythms in Expression of Genes of Hepatic Lipid Metabolism in Atlantic Salmon (Salmo salar L.)
Source: PLoS One. 2014 Sep 3;9(9):e106739. doi: 10.1371/journal.pone.0106739 (PMC4153669; doi:10.1371/journal.pone.0106739)
Supplement: Table S2 — Primer pairs and sequences for Rev-erb1α identification including primer name, purpose, sequence and annealing temperature. (DOCX) [file pone.0106739.s004.docx]

**Supplementary Table 2**.- Primer pairs and sequences for *Rev-erb1α* identification including primer name, purpose, sequence and annealing temperature.

| **Name** | **Purpose** | **Sequence 5’-3’** | **Tm (˚C)** |
| --- | --- | --- | --- |
| *NR1D1 5’R1* | RACE-PCR | GCCCCAGTTGTCCACCTCTCCGTTATGT | 60 ° |
| *NR1D1 5’R2* | RACE-PCR | AATGGCGGGCTTTGGGTGGATG | 60 ° |
| *NR1D1 3’F1* | RACE-PCR | TACCCCCAAGACGAACCCAACA | 60 ° |
| *NR1D1 3’F2* | RACE-PCR | GGGAGGCTTGCTAGACACCAT | 60 ° |
| *NR1D1_full_F1* | Full length outer PCR | AGGCCGACTTGGAAACTGC | 57 ° |
| *NR1D1_full_R1* |  | GTCTATTGGCCTTACCCCTATCA | 57 ° |
| *NR1D1_full_F2* | Full length inner PCR | GTTCAGACCTGCACCGATAGAGC | 62 ° |
| *NR1D1_full_R2* |  | TAGCCGCCCAACCACCACTGTC | 62 ° |
