# Supplementary material for: Daily Rhythms in Expression of Genes of Hepatic Lipid Metabolism in Atlantic Salmon (Salmo salar L.)
Source: PLoS One. 2014 Sep 3;9(9):e106739. doi: 10.1371/journal.pone.0106739 (PMC4153669; doi:10.1371/journal.pone.0106739)
Supplement: Table S3 — Primers used for qRT-PCR. (DOCX) [file pone.0106739.s005.docx]

| **Supplementary Table 3**.- Primers used for qRT-PCR. | **Gene** | **Primer Sequence (5’-3’)** | **Amplicon (bp)** | **Tm (˚C)** | **Accession no** |
| --- | --- | --- | --- | --- | --- |
| **CLOCK** | *Bmal1* | F: GCCTACTTGCAACGCTATGTCC | 90 | 64˚ | DY 735402^a^ |
|  |  | R: GCTGCGCCTCGTAATGTCTTCA |  |  |  |
|  | *Clock* | F: AGAAATGCCTGCACAGTCGGAGTC | 196 | 64˚ | CA 038738^a^ |
|  |  | R: CCACCAGGTCAGAAGGAAGATGTT |  |  |  |
|  | *Per 1* | F: AGGGGGTCATGCGGAAGGGGAAGT | 150 | 66˚ | Unpublished |
|  |  | R: TGGGCCACCTGCATGGGCTCTGT |  |  |  |
|  | *Per 2* | F: GCTCCCAGAATTCCTAGTGACAAG | 88 | 60˚ | FM877775^a^ |
|  |  | R: GAACAGCCCTCTCGTCCACATC |  |  |  |
|  | *Rev-erb1α* | F: CCCCCAAGACGAACCCAACAAGAC | 194 | 61˚ | 1714461^a^ |
|  |  | R: AGAGGGAGGCAAAGCGCACCATTA |  |  |  |
| **Lipid Transcription factors** | *Srebp1* | F: gccatgcgcaggttgtttcttca | 151 | 63˚ | TC148424^b^ |
|  |  | R: Tctggccaggacgcatctcacact |  |  |  |
|  | *Srebp2* | F: TCGCGGCCTCCTGATGATT | 147 | 63˚ | TC166313^b^ |
|  |  | R: AGGGCTAGGTGACTGTTCTGG |  |  |  |
|  | *Lxr* | F: GCCGCCGCTATCTGAAATCTG | 210 | 58˚ | FJ470290a |
|  |  | R: CAATCCGGCAACCAATCTGTAGG |  |  |  |
|  | *Pparα* | F: TCCTGGTGGCCTACGGATC | 111 | 60˚ | DQ294237^a^ |
|  |  | R: CGTTGAATTTCATGGCGAACT |  |  |  |
|  | *Pparγ* | F: CATTGTCAGCCTGTCCAGAC | 144 | 60˚ | AJ416951^a^ |
|  |  | R: TTGCAGCCCTCACAGACATG |  |  |  |
| **Cholesterol Metabolism** | *Mev* | F: CCCTTAATCAGGGTCCCAAT | 247 | 60˚ | DW005667^a^ |
|  |  | R: GGTGCTGGTTGATGTCAATG |  |  |  |
|  | *Dhcr7* | F: CTTCTGGAATGAGGCATGGT | 230 | 60˚ | TC99602^b^ |
|  |  | R: ACAGGTCCTTCTGGTGGTTG |  |  |  |
|  | *Ipi* | F: ACAGCCCTATGGTTATGTGTCATCTC | 230 | 60˚ | CK875291^a^ |
|  |  | R: CAAGGTGAGGCGAATGTTTGAAC |  |  |  |
|  | *Abca1* | F: GGACGAACCCTGTGTCTGTT | 212 | 60˚ | EG836783^a^ |
|  |  | R: ATTTGCATTGCGTTTCAGTG |  |  |  |
|  | *Cyp71α* | F: TGGAGATCTTCCGGCACTCT | 101 | 60˚ | BT059202^a^ |
|  |  | R: CAGGTGTCCTTGGGAATGGA |  |  |  |
| **Fatty acid Biosynthesis** | *D5fad* | F: GTGAATGGGGATCCATAGCA | 192 | 56˚ | AF478472^a^ |
|  |  | R: AAACGAACGGACAACCAGA |  |  |  |
|  | *D6fad* | F: GTGAATGGGGATCCATAGCA | 192 | 60˚ | GU294488^a^ |
|  |  | R: AAACGAACGGACAACCAGAC |  |  |  |
|  | *Elovl2* | F: CGGGTACAAAATGTGCTGGT | 145 | 60˚ | TC91192^b^ |
|  |  | R: TCTGTTTGCCGATAGCCATT |  |  |  |
|  | *Elovl5a* | F:ACAAGACAGGAATCTCTTTCAGATTAA | 137 | 60˚ | AY170327^a^ |
|  |  | R: TCTGGGGTTACTGTGCTATAGTGTAC |  |  |  |
|  | *Hmgcr* | F: CCTTCAGCCATGAACTGGAT | 224 | 60˚ | DW561983a |
|  |  | R: TCCTGTCCACAGGCAATGTA |  |  |  |
|  | *Fas* | F: ACCGCCAAGCTCAGTGTGC | 212 | 60˚ | CK876943a |
|  |  | R: CAGGCCCCAAAGGAGTAGC |  |  |  |
| **FA Catab** | *Cpt-1* | F: CCTGTACCGTGGAGACCTGT | 212 | 60° | AM230810^a^ |
|  |  | R: CAGCACCTCTTTGAGGAAGG |  |  |  |
|  | *Aco* | F: AAAGCCTTCACCACATGGAC | 230 | 60° | TC49531^b^ |
|  |  | R: TAGGACACGATGCCACTCAG |  |  |  |
| **Lipoprotein metaolism** | *ApoA1* | F: CCATCAGCCAGGCCATAAA | 73 | 60° | CB506105^a^ |
|  |  | R: TGAGTGAGAAGGGAGGGAGAGA |  |  |  |
|  | *ApoB* | F: AGCCTTCGATGCTGTCGGCCA | 153 | 60° | TC79364^b^ |
|  |  | R: AGGAGCACAGGCAGGGTGGTT |  |  |  |
|  | *ApoCII* | F: GGAACCAGTCGCAGATGTTGA | 145 | 60° | DN047858^a^ |
|  |  | R: TGAGGACATTCGTGGCCTTC |  |  |  |
|  | *Ldlr* | F: GCATGAACTTTGACAATCCAGTGTAC | 78 | 60° | AJ003118^a^ |
|  |  | R: TGGAGGAGTGCCTGCTGATAT |  |  |  |
|  | *El* | F: CCGGTGCTGCTGGAGGAAGC | 378 | 60° | NM_001140535^a^ |
|  |  | R: CGACATGCAGGTCATCGGT |  |  |  |
|  | *Lpla* | F: TGCTGGTAGCGGAGAAAGACAT | 114 | 60° | BI468076^a^ |
|  |  | R: CTGACCACCAGGAAGACACCAT |  |  |  |
|  | *Lplb* | F: GGCAGCCCTACATGATAACC | 172 | 60° | TC67836^b^ |
|  |  | R: TCTGTCCAAAGCCACTCACA |  |  |  |
|  | *Lplc* | F: AGGGCGTTAATCCATGTCAG | 223 | 60° | TC84899^b^ |
|  |  | R: GACCTTTCAAAAGGGCATGA |  |  |  |
| HK | *Efa* | F: CTGGAGACGCTGCTATTGTTG | 175 | 60° | AF321836^a^ |
|  |  | R: GACTTTGTGACCTTGCCGCTTGAG |  |  |  |

^a^GenBank (http://www.ncbi.nlm.nih.gov/)

^b^Atlantic salmon Gene Index (http://compbio.dfci.harvard.edu/tgi/)
